# Detection of nasopharyngeal carcinoma in Morocco (North Africa) using a multiplex methylation-specific PCR biomarker assay

**DOI:** 10.1186/s13148-015-0119-8

**Published:** 2015-08-22

**Authors:** Imran Nawaz, Khalid Moumad, Debora Martorelli, Moulay Mustapha Ennaji, Xiaoying Zhou, Zhe Zhang, Riccardo Dolcetti, Meriem Khyatti, Ingemar Ernberg, Li-Fu Hu

**Affiliations:** Department of Microbiology, Tumor and Cell Biology, Karolinska Institutet, Box 280, Stockholm, SE-17177 Sweden; Department of Microbiology, Faculty of Life Sciences, University of Balochistan, Quetta, Pakistan; Department of Molecular Genetic Epidemiology, German Cancer Research Center (DKFZ), 69120 Heidelberg, Germany; Oncovirology Laboratory, Institut Pasteur du Maroc, 20360 Casablanca, Morocco; Cancer Bio-Immunotherapy Unit Centro di Riferimento Oncologico IRCCS - National Cancer Institute, Via Franco Gallini, 233081 Aviano, PN Italy; University Hassan II, Faculty of Sciences and Techniques, Mohammedia - Casablanca, Laboratory of Virology, Microbiology and Quality/ETB, Mohammedia, , BP 146, 20650 Morocco; Department of Orolaryngology – Head and Neck Surgery, First Affiliated Hospital of Guangxi Medical University, Guangxi, People’s Republic of China

**Keywords:** NPC, Morocco, DNA methylation, TSGs, Bisulfite conversion, Biomarker, Early diagnosis, Screening, MMSP

## Abstract

**Background:**

Silencing of tumor suppressor genes (TSGs) or activation of oncogenes by, e.g., aberrant promoter methylation, may be early events during carcinogenesis. The methylation status of such genes can be used for early detection of cancer. We are pursuing this approach in our efforts to develop markers for early detection and follow-up of nasopharyngeal carcinoma (NPC). We set out to develop this approach to allow identification of NPC from Morocco and then also compared with NPC samples from different geographical locations and different ethnicity with different NPC incidences, Epstein-Barr virus (EBV) prevalence, and environments.

**Results:**

By multiplex methylation-specific PCR (MMSP), multiple relevant genes can be detected simultaneously, to achieve high sensitivity and specificity. The strong association of EBV with NPC is also very useful in such an approach. We have initially screened for 12 potential marker genes including EBV genes coding for EBV nuclear antigen 1 (*EBNA1*) and latent membrane protein-1 (*LMP1*) and ten potential TSGs obtained from previously published data. The resulting assay included *EBNA1*, *LMP1*, and three cellular TSGs: *ITGA9*, *RASSF1A*, and *P16*. We evaluated this assay on 64 NPC patient biopsies from Morocco, Italy, and China compared to deoxyribonucleic acid (DNA) from 20 nasopharyngeal control tissues. In the Moroccan NPC cohort (*n* = 44), prevalence of the *EBNA1* gene showed the highest sensitivity (36/44; 82 %) with 94 % specificity. Out of eight (18 %) *EBNA1* negative Moroccan samples, only three were positive for at least one methylated cellular gene. By detection of cellular marker genes, the sensitivity increased from 82 to 89 % (39/44). In the whole material of 64 biopsies from three geographical locations, at least any one marker (viral or cellular) could be detected in 91 % of biopsies with 90 % specificity. In a pilot evaluating assay performance on serum DNA from NPC and controls including samples from Italy (*n* = 11) and China (*n* = 5), at least any one marker from the MMSP assay could be detected in 88 %, but the specificity was only 50 %.

**Conclusions:**

An MMSP assay has the potential for detection of NPC by screening in high-risk populations. Serum-derived DNA seems not as good as earlier published NPC swab DNA for screening purpose.

## Background

Nasopharyngeal carcinoma (NPC) is a malignant tumor that arises in the mucosal epithelium of the posterior wall of the nasopharynx [1]. Its clinical presentation, epidemiology, and histopathology are different from other squamous cell carcinomas of the head and neck [2]. NPC has a marked ethnic and geographic distribution. Specifically, its most common form, World Health Organization (WHO) type III, is highly prevalent in southern China, Southeast Asia, North Africa, and Greenland and is strongly associated with the Epstein-Barr virus (EBV) [3–7]. In Morocco, NPC is the most frequent tumor of the head and neck region accounting for 7–12 % of all cancers in men [8, 9]. Interestingly, NPC has also been reported to be the most common neoplasia of the nasopharynx and respiratory tract in children in Morocco and Tunisia accounting for 5–20 % of childhood malignancies [10, 11, 9]. This is even higher than its incidence in the high endemic NPC areas in China, where it is 0.1 % in children [12, 13]. Differences in NPC incidence are also reported in the same geographical locations between members of different ethnic groups [14, 15]. Certain dietary habits such as consumption of salted fish and preserved food containing volatile nitrosamines are also reported to play some role in the etiology of NPC [16–18]. Regardless of incidence and geographical distribution, its development has been attributed to an interaction of multiple factors: environmental including EBV infection and genetic factors [19, 20]. Chromosomal abnormalities and aberrant promoter hypermethylation show a similar pattern in different geographical regions according to one study so far, supporting a common carcinogenic pathway to NPC irrespective of region [21].

Approximately 70 % of NPC is diagnosed in advanced stages when treatment results are unsatisfactory. This is largely due to the non-specific local symptoms [3, 22–24]. Diagnosis of NPC at stages I or II is associated with a high survival rate (on average 95 %), whereas the survival rate is just above 50 % when diagnosis is made late at stages III or IV [25]. Because of a higher cure rate for early-stage NPC, the concept of screening for the disease has an intuitive appeal. Further development of therapies is necessary but not sufficient to improve the survival of NPC patients [26]. The development of reliable, non-invasive, and cost-effective early-detection methods for NPC is a high priority. Application of tumor markers thus provides a relevant approach to achieve early detection of NPC. The rapid acquisition of fully sequenced cancer genomes will be one powerful tool to identify biomarker candidates for early detection, subtyping, and cancer screening [27].

It has been suggested that cancer can be initiated by epigenetic changes before any mutations take place. This epigenetic progenitor hypothesis is supported by experimental data on, e.g., colon cancer [28]. This makes epigenetic marks highly interesting for early diagnosis of cancer [29, 30]. In the case of NPC, latent EBV infection may drive epigenetic changes in normal epithelial cells to promote tumorigenesis [31]. But genetic alterations including deoxyribonucleic acid (DNA) methylation of cyclin-dependent kinase inhibitor 2A (P16) and RASSF1A loci have been reported to occur in premalignant nasopharyngeal epithelium even prior to EBV infection [32]. Aberrant methylation of tumor suppressor genes (TSGs), e.g., P16, is a frequent event in NPC [33]. DNA methylation also plays an important role in the maintenance of specific EBV latency programs in the NPC cells [34, 31, 35]. Aberrant methylation of both viral and cellular genes may thus be involved in the transformation and progression of nasopharyngeal epithelial cells into malignant ones [36–38]. Screening for DNA hypermethylated marker genes, e.g., TSGs, has a great potential to be used as an assay for early detection of NPC [39]. Moreover, the EBV-host epigenetic interplay and the reversible nature of epigenetic mechanism of gene regulation in NPC make such genes interesting also in the context of NPC therapy and prevention [40–42]. Recently, it was demonstrated that gastric carcinoma associated with EBV is strongly hypermethylated (as a whole tumor subgroup) compared to EBV-negative gastric carcinoma [43–45].

Several techniques for analyzing DNA methylation status have been developed, each one with advantages and limitations. As an example, methylation-specific PCR (MSP) allows detection of one methylated gene copy among 1000 unmethylated copies [46] but it can only be applied to one gene at a time. For high-throughput methylation studies, state-of-the-art equipment and qualified bioinformatics are required. Until now, this makes such techniques unsuitable at the clinical level [47]. Due to this, we previously developed a simple and rapid PCR-based assay designated multiplex methylation-specific PCR (MMSP) which could detect the methylation status of multiple markers in a single reaction simultaneously [48].

When applied to samples from regions with a lower NPC risk than China like Morocco, North Africa (medium risk), its performance decreased, and thus, we tried to modify the assay to obtain good specificity and sensitivity for tumors in such areas. This modified MMSP assay includes, as before, prevalence of the EBV nuclear antigen 1 (*EBNA1*) (not methylated), important for distinguishing between EBV-positive and EBV-negative subtypes of NPC or non-cancerous control samples. The expression level of EBV-encoded latent membrane protein-1 (*LMP1*), which is an EBV oncoprotein, expressed in approximately 65 % of EBV-positive NPC patients and was regulated by promoter methylation [49]. Outcome of *LMP1* in the MMSP panel would reflect *LMP1*-expression status. In addition to the EBV genes, three TSGs namely integrin, alpha 9 (*ITGA9*); Ras association (RalGDS/AF-6) domain family member 1 (*RASSF1A*); and *P16* were included after screening the methylation status of ten putative TSGs selected from published data (including our own; *ITGA9*; *RASSF1A*; *P16*; death-associated protein kinase (*DAPK*); wingless-type MMTV integration site family, member 7A (*WNT7A*); checkpoint with forkhead and ring finger domains (*CHFR*); cytochrome b5 reductase 2 (*CYB5R2*); WNT inhibitory factor 1 (*WIF1*); PR domain containing 2, with ZNF domain (*RIZ1*); and follistatin-like 1 (*FSTL1*)) on NPC tumor biopsies and evaluating their individual sensitivity and specificity.

MMSP allows analyses of the methylation status of multiple genes in a single reaction. This is cost-effective and could be suitable for detection of NPC in patients from different geographical, environmental, and genetic backgrounds and of different subtypes (e.g., EBV-positive or EBV-negative). Early detection will be an instrumental step improving therapeutic results. For this, invasive biopsy sampling will not be suitable on a large scale so less invasive sources of DNA have to be sought. We earlier evaluated nasopharyngeal secretions (swabs) and now made a small pilot evaluating the assay performance on serum DNA from NPC and controls. However, specificity was rather low in serum and one can question its representation of tumor specificity. Thus, NPC swab DNAs are to be preferred while using serum-derived DNA could possibly be improved.

## Results

### Identification of additional candidate methylated markers for NPC using MSP

All the DNAs from the NPC and normal biopsy samples from Morocco were screened using MSP for 12 markers (including the markers used in the MMSP assay published previously [48] comprising *EBNA1*, *LMP1*, *RASSF1A*, and *DAPK* but also 8 new markers including *ITGA9*, *P16*, *WNT7A*, *CHFR*, *CYB5R2*, *WIF1*, *RIZ1*, *FSTL1*). The results are summarized in Table 1. Of the Moroccan samples, 36/44 (82 %) were positive for EBNA1 while the remaining 8 samples (18 %) were negative. This result on Moroccan NPC patients differed from that of southern China where the common type of undifferentiated NPC is strongly associated with EBV [15, 50–57]. *RASSF1A* was positive in 29/44 as the most sensitive marker (66 %) after *EBNA1*. Among the previously published MMSP assay marker genes, *DAPK* showed the lowest sensitivity 11/44 (25 %) in Moroccan NPC DNA samples, and the specificity was 13/18 (72 %) when comparing to non-cancerous nasopharyngeal epithelium from the same geographic location. These results motivated an effort to replace *DAPK* with a marker with higher sensitivity and specificity. Eight genes were selected from published literature and screened on NPC samples and controls from Morocco. *ITGA9* and *P16* were the two with the highest specificity (100 %) and better sensitivity than *DAPK* (Sn 22/44, 50 % and Sn 20/44, 45 %, respectively). From these results, it was apparent, as expected, that a single gene cannot be used for screening/early detection. Therefore, we set out to explore combinations of several genes and modify our previously published MMSP assay to improve it so that it could be applied on samples from different geographical locations, including also EBV-negative NPC.

**Table 1:** Sensitivity and specificity of markers in DNAs from NPC samples (*n* = 44) and non-cancerous controls (*n* = 18) from Morocco using MSP

Marker gene	Sensitivity %^a^	Specificity %^b^
*EBNA1*	82 (36/44)	94 (17/18)
U-*LMP1*	59 (26/44)	94 (17/18)
M-*RASSF1A*	66 (29/44)	94 (17/18)
M-*DAPK*	25 (11/44)	72 (13/18)
M-*ITGA9*	50 (22/44)	100 (18/18)
M-*P16*	45 (20/44)	100 (18/18)
M-*WNT7A*	69 (11/16)	80 (4/5)
M-*CHFR*	40 (16/40)	67 (4/6)
M-*CYB5R2*	47 (17/36)	75 (6/8)
M-*WIF1*	100 (10/10)	25 (2/8)
M-*RIZ1*	0 (0/4)	100 (3/3)
M-*FSTL1*	57 (4/7)	67 (6/9)

^a^Sensitivity = number of positive cases in NPC patients/total number of NPC cases tested

^b^Specificity = (total number of tested non-cancerous controls − number of positive cases in tested non-cancerous controls)/total number of tested non-cancerous controls

A comparison of sensitivity and specificity of *DAPK* with a combination of *ITGA9* and *P16* (where either or both of them were methylated) showed an increase in sensitivity and specificity (Table 2). Either *ITGA9* (*n* = 11) or *P16* (*n* = 9) or both (*n* = 11) were methylated in 70 % (31/44) DNA samples from Morocco with 100 % (18/18) specificity.

**Table 2:** Comparison of sensitivity and specificity of *DAPK* and combination of *ITGA9* and *P16* (where either or both of them were methylated) on NPC samples (*n* = 44) and non-cancerous controls (*n* = 18) from Morocco

Marker gene	Sensitivity	Specificity
M-*DAPK*	25 % (11/44)	72 % (13/18)
M-*ITGA9*/M-*P16*	70 % (31/44)	100 % (18/18)

### Development of modified multiplex methylation-specific PCR and screening NPC biopsy and control samples from Morocco

On the basis of MSP results, a modified MMSP assay was developed which comprised *EBNA1* and *LMP1* as before, but *DAPK* was replaced with *ITGA9* and *P16* based on their 100 % specificity and higher sensitivity. The housekeeping gene *β-ACTIN* as DNA-control was used alternatingly with glyceraldehyde-3-phosphate dehydrogenase (*GAPDH*) as internal loading controls and quality controls for bisulfite conversion of DNA. The modified MMSP was used to screen 64 NPC patient biopsies with 16 corresponding serum samples from Morocco, Italy, and China compared to DNA from 20 nasopharyngeal tissues and 8 serum samples from persons without cancer.

**Fig. 1:**
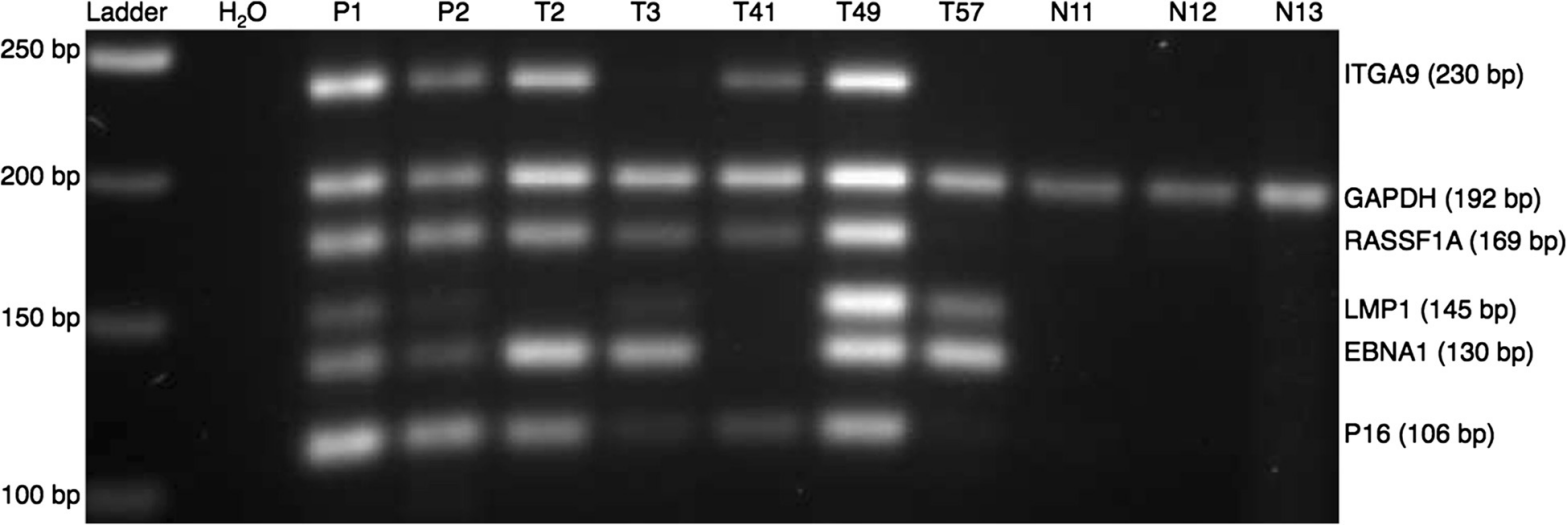
MMSP assay applied to DNAs from NPC tissue and non-cancerous control from Morocco. A mixture of cell line DNA from CNE1 and Namalwa was used as a positive control (see the “Methods” section) as follows: P1: 40 ng CNE1 and Namalwa DNA in 1:2 ratio; P2: 5 ng CNE1 and Namalwa DNA in 1:2 ratio. Water was used as a blank control. *T* NPC tissue, *N* non-cancerous controls

We first analyzed only the 36 *EBNA1* positive NPC samples from Morocco (Fig. 1). The marker gene pattern in NPC DNA was clearly different from that of non-cancerous controls. In the *EBNA1* positive samples, *RASSF1A* was detected in 28/36 and thus was the most sensitive marker (78 %). Marker genes *ITGA9* and *P16* showed 56 and 50 % sensitivity, respectively, but both of them were 100 % specific. The MMSP results in the *EBNA1* positive samples are summarized in Table 3.

**Table 3:** Sensitivity and specificity of markers in *EBNA1* positive NPC samples (*n* = 36) and non-cancerous controls (*n* = 18) from Morocco using modified MMSP assay

Marker gene	Sensitivity %(positive cases/total)	Specificity % (total cases − positive cases/total control cases)
U-*LMP1*	72 (26/36)	94 (17/18)
M-*ITGA9*	56 (20/36)	100 (18/18)
M-*RASSF1A*	78 (28/36)	94 (17/18)
M-*P16*	50 (18/36)	100 (18/18)

In Table 4, we show the co-occurrence of methylated marker genes in the *EBNA1* positive samples with our MMSP marker panel (i.e., *RASSF1A*, *LMP1*, *ITGA9*, and *P16*). The majority of the NPC biopsies were positive for more than one marker in addition to *EBNA1*. No methylated marker gene could be detected in one *EBNA1* positive NPC sample (3 %). The remaining NPC samples (97 %; 35/36) were positive for at least any one marker in addition to *EBNA1*, whereas all but one (17/18; 94 %) of the non-cancerous samples were negative with the criterion of *EBNA1* plus at least one methylation marker. The sensitivity and specificity with at least any two markers were, respectively, 83 and 94 % that is also applicable for such an assay. The positive predictive value (PPV) and negative predictive value (NPV) in samples from Morocco were 97 and 94 %, respectively.

**Table 4:** Different combinations of modified MMSP markers observed in NPC samples selected on the basis that they were EBV (*EBNA1*)-positive (*n* = 36) and non-cancerous controls (*n* = 18) from Morocco

Marker genes/combinations	1	2		3				4			5
*EBNA1*	+	+	+	+	+	+	+	+	+	+	+
U-*LMP1*	-	+	-	+	+	-	-	+	+	-	+
M-*ITGA9*	-	-	-	+	-	+	-	+	-	+	+
M-*RASSF1A*	-	-	-	-	+	+	+	+	+	+	+
M-*P16*	-	-	+	-	-	-	+	-	+	+	+
NPC samples (*n* = 36)	1	4	1	2	3	2	2	6	5	4	6
Control (*n* = 18)	0	0	0	0	1	0	0	0	0	0	0
EBNA1 + at least 3 methylation markers								Sn: 58 % (21/36)			
								Sp: 100 % (18/18)			
EBNA1 + at least 2 methylation markers				Sn: 83 % (30/36)							
				Sp: 94 % (17/18)							
EBNA1 + at least 1 methylation marker		Sn: 97 % (35/36)									
		Sp: 94 % (17/18)									

EBV *EBNA1* could not be detected in 18 % (8/44) of our NPC biopsy samples from Morocco. These are representatives of subtypes of NPC most commonly found in the non-endemic regions, e.g., in Europe and USA. Only in three out of these eight *EBNA1* negative NPC biopsy samples could we detect at least one methylated cellular marker gene. One sample showed methylated *ITGA9*, another one *P16*, and the third methylated *ITGA9*, *P16*, and *RASSF1A*. Taken together, irrespective of *EBNA1* status, 89 % (39/44) of Moroccan samples were positive with at least any one positive viral or cellular marker.

### Analyzing tumors and sera from Chinese and Italian NPC and controls with modified MMSP

**Fig. 2:**
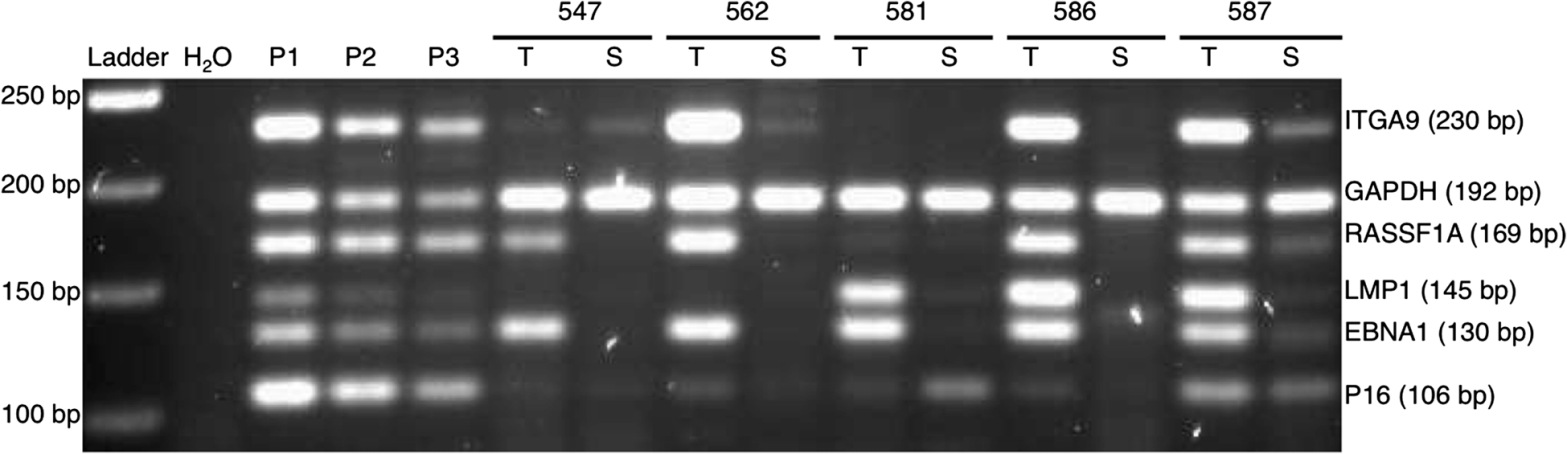
MMSP assay applied to DNAs from matched NPC tissue and serum from China. A mixture of cell line DNA from CNE1 and Namalwa was used as a positive control as follows: P1: 40 ng CNE1 and Namalwa DNA in 1:2 ratio; P2: 10 ng CNE1 and Namalwa DNA in 1:2 ratio; P3: 5 ng CNE1 and Namalwa DNA in 1:2 ratio. Water was used as a blank control. *T* NPC tissue, *S* serum

**Fig. 3:**
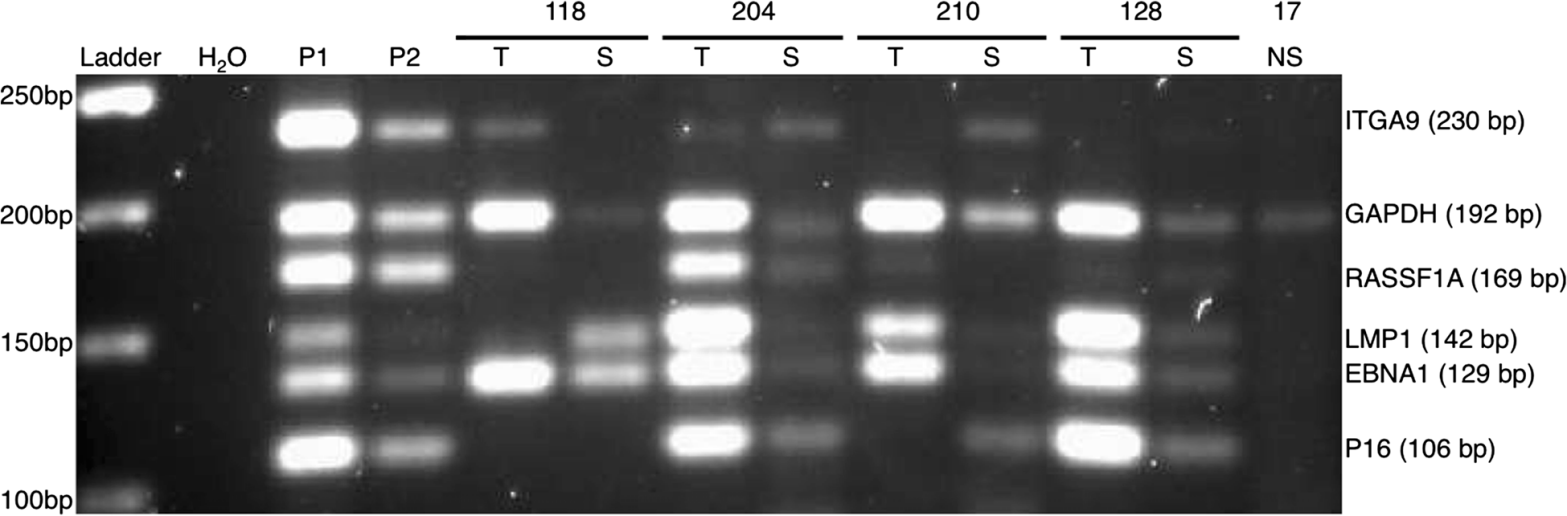
MMSP assay applied to DNAs from matched NPC tissue, serum, and non-cancerous serum from Italy. A mixture of cell line DNA from CNE1 and Namalwa was used as a positive control as follows: P1: 40 ng CNE1 and Namalwa DNA in 1:2 ratio; P2: 5 ng CNE1 and Namalwa DNA in 1:2 ratio. Water was used as a blank control. *T* NPC tissue, *S* serum, *NS* non-cancerous serum

We applied the MMSP to a subset of our samples irrespective of *EBNA1* status including two independent small cohorts from China and Italy on tumor DNA samples with paired matched serum samples (Table 5). One hundred percent (5/5) of tumor DNA from tumor and 80 % (4/5) of corresponding NPC sera were positive in Chinese samples (Fig. 2). In the Chinese cohort, we had control DNA only from two biopsies, and one of them was negative for all the MMSP markers. In DNA samples from Italy, we could identify 93 % (14/15) as NPC with at least any one MMSP marker (Fig. 3). Ninety-one percent (10/11) of serum samples from Italy were positive as NPC-related. In the Italian cohort, we also had eight control DNA samples from sera. The specificity among these samples was also as low as 50 %. Remarkably, the pattern of MMSP markers observed in biopsy samples did not match with the patterns found in the corresponding serum samples from both China and Italy.

**Table 5:** Comparison of sensitivity and specificity of previous and modified MMSP markers on DNAs from NPC and control samples from Morocco, Italy, and China using at least any one viral or cellular marker (irrespective of *EBNA1* status)

Origin	DNA source	Previous panel of markers		Modified panel of markers	
		Sensitivity	Specificity	Sensitivity	Specificity
Morocco	Tumor	84 % (37/44)	67 % (12/18)	89 % (39/44)	94 % (17/18)
Italy	Tumor			93 % (14/15)	NA
	Serum			91 % (10/11)	50 % (4/8)
China	Tumor			100 % (5/5)	50 % (1/2)
	Serum			80 % (4/5)	NA
Total	Tumor	84 % (37/44)	67 % (12/18)	91 % (58/64)	90 % (18/20)
	Serum			88 % (14/16)	50 % (4/8)

*NA* not applicable

We tested the modified MMSP assay on DNAs from 64 NPC biopsies from three different geographical regions (Morocco 44, Italy 15, and China 5), 16 corresponding NPC sera (Italy 11 and China 5), 20 control biopsies (Morocco 18 and China 2), and 8 control sera from Italy. In DNAs from biopsies, we could detect at least one marker in 91 % (58/64) at 90 % (18/20) specificity. In the smaller cohort of serum samples, at least one marker could be detected in 88 % (14/16) of the samples at 50 % (5/10) specificity.

## Discussion

The unique features of NPC, its regional incidence, and its strong association with EBV [58] have led to an intense effort to establish the role of EBV in its pathogenesis, to find possible susceptibility genes and regional environmental factors that could lead to improved diagnosis, treatment, prognostic tools, and possibly prevention. Well over a 100 cellular genes and several EBV genes have been implicated in the disease progression. The number of genes deregulated by epigenetic mechanisms, primarily promoter methylation, is steadily growing.

Our present knowledge of cancer genomes can be translated into improved methods to reduce cancer incidence and related deaths [27]. A panel of three to four marker genes could define an abnormality in 70–90 % of each cancer type studied by detection of their aberrant methylation [59]. It has also been observed that the EBV methylome in EBV-positive cancer cells is significantly different compared to that of EBV-carrying cells representing non-tumorigenic human B-cell-derived lymphoblastoid cell lines [59]. Thus, epigenetic signatures from both human cellular genes and EBV genes may pose an ideal combination to detect NPC or discriminate NPC from malignancies of other origins [60]. A combination of methylation-regulated TSGs including *RASSF1A*, *P16*, and EBV-based markers could serve as a complementary test for the early detection of NPC [47, 61].

Based on our MMSP results on all the 44 NPC samples from Morocco, five different patterns were seen: only *EBNA1* positive; *EBNA1* + *LMP1* positive; positive for any methylated TSG, i.e., *RASSF1A*, *ITGA9*, or *P16*; *EBNA1* negative; and negative for all MMSP markers and *EBNA1*. In this study, 36/44 (82 %) Moroccan NPC samples were positive for *EBNA1*. Among eight *EBNA1* negative samples, five samples (11 %) were negative for all MMSP markers except DNA control *GAPDH*. The prevalence of EBV in NPC has been reported to differ between different geographical locations, in China 100 % [48], Indonesia 92.5 % [47], and Tunisia 100 % [61]. In Europe and the US, the prevalence of EBV-positive cases is much lower, estimated to 5–10 %, and these NPCs are mostly WHO type I. EBV may act as an epigenetic driver in NPC tumorigenesis [34] by genome-wide hypermethylation [62, 63]. EBV-negative NPC has been reported to show lower frequencies of TSG promoter hypermethylation as compared to EBV-positive NPC [34]. In our case, the prevalence of EBV in NPC samples from Morocco was 82 %. Among the *EBNA1* negative samples (*n* = 8), methylated DAPK was not detected, but one sample was positive only for *P16* and another such sample was positive for *ITGA9*. A third *EBNA1* negative sample was positive for *ITGA9*, *RASSF1A*, and *P16*. These samples might represent EBV-negative NPC. The possibility of diagnostic errors or non-representative sampling in the EBV-negative NPC cannot be excluded. Nevertheless, at least any one viral or cellular marker of modified MMSP could be detected in 89 % of all cases from Morocco.

Applying the previously published MMSP panel of markers on all the Moroccan NPC samples (irrespective of *EBNA1* presence or absence), at least any one marker could be detected positive in 84 % (37/44) with 67 % (12/18) specificity. In the previous publication, the sensitivity was 98 % (48/49) with 100 % (20/20) specificity when the assay was applied on Chinese NPC. This difference in the sensitivity and specificity of the same panel of markers might be due to differences in ethnicity, environmental factors, and EBV infection rates between these geographical regions. With the new, modified MMSP applied on the Moroccan samples, any one positive marker could be detected in 89 % (39/44) with 94 % (17/18) specificity (Table 5).

DNA coding for *EBNA1*, the nuclear household protein of EBV, was used as a marker for EBV prevalence. As the part of the *EBNA1* gene amplified by PCR is present only once in an EBV genome, it has the potential of quantifying EBV DNA load in, e.g., NPC swab or serum, using a one/haplome EBV-positive cell as reference (Namalwa). Few malignancies express the EBV-encoded oncoprotein, *LMP1*. The *LMP1* primers used in the assay specially amplified bisulfite-converted unmethylated alleles. Judging the *LMP1* methylation status in a specific NPC adds to the specificity of the MMSP and potentially provides information about the prognosis of NPC, known to be affected by LMP1 [49]. Out of 36 *EBNA1* positive NPC samples, 26 samples (72 %) were also positive for unmethylated *LMP1* which is quite consistent with our previous study (Table 3) [49].

The primers for *ITGA9*, *RASSF1A*, and *P16* were designed to amplify methylated alleles only. Among these, *ITGA9* is a component of the α9β1 integrin receptor that plays an integral role in different signal transduction pathways controlling cellular proliferation and differentiation. In *ITGA9* knockout mice, abnormal proliferation and differentiation of keratinocytes suggest its role in these cellular processes [64]. Here, we showed that *ITGA9* is methylated in NPC. *RASSF1A* is also a strong candidate TSG for NPC. The *RASSF1A* protein could interact with DNA repair system and also induce cell-cycle arrest. It has been shown to be frequently methylated in NPC biopsies, and the aberrant methylation is tightly correlated with loss of expression of *RASSF1A* in NPC [65, 66]. P16 inactivates the cyclin D-cyclin-dependent kinase 4 (or 6) complex resulting in the inactivation of retinoblastoma protein and thus blocks the transcription of important cell-cycle regulatory proteins and results in cell-cycle arrest. It is a major target in tumorigenesis and is altered in multiple primary tumors [67]. Promoter hypermethylation of *P16* is frequent in NPC [68, 69]. Most importantly, the loss of *P16* may be an early event in cancer progression [67] which made *P16* a good candidate for our MMSP assay.

The strong association of EBV with undifferentiated NPC prevalent in high-endemic areas makes EBV a good marker for screening in a high-risk population. However, EBV-based markers are not enough for identification of NPC types that are not associated with EBV, which are found in intermediate and prevalent in low-risk areas. The inclusion of cellular marker genes in an assay like MMSP will be very important for identifying EBV-negative NPC cases. *EBNA1* was not detected in 14 % (8/44) of our biopsy samples from Morocco. In three out of these eight samples, we could detect the methylation of at least any one methylated cellular gene. This shows the importance of cellular genes in the MMSP assay for the identification of EBV-negative samples but also that we had been unable to find the most suitable marker genes for such cases. Alternatively, the five totally negative biopsies might have been non-representative of NPC. In future studies, such results demand further validation by pathology. The presence of cellular TSGs in the MMSP assay increased the sensitivity of the assay from 84 to 89 % without affecting the specificity (94 %).

The low level of EBV DNA persists throughout life in infected B cells in all blood-perfused tissues of EBV-infected people (i.e., more than 90 % of the adult population globally) [70, 71]. The low level of EBV DNA is also found in saliva in some of these healthy carriers. Detection of EBV DNA, e.g., with the *EBNA1* gene, thus poses a slight risk of false positives using nasopharyngeal swabs, saliva, blood, plasma, and even serum. The levels are usually so low that they end up below cutoff also in regular PCR assays. This is an additional argument why one should not only rely on EBV genes in future screening protocols. In this study, we could identify 84 % (10/11) of NPC with only 50 % (5/10) specificity when sera were used as a DNA source. This might be because the serum contains few circulating tumor cells (CTCs) and cell-free tumor DNA (cftDNA) while the majority of the cells in the serum are lymphocytes. The possibility of using serum for the early detection of cancer can improve with improvements of CTC and cftDNA isolation and enrichment. With high-throughput (HTP) methods for the isolation or enrichment of CTC or cftDNA, specificity and sensitivity might be improved for serum samples. Our earlier work showed that DNA swabs with nasopharyngeal secretion were a much better source of DNA for screening EBNA1 and at least any one additional marker could be detected in 98 % (48/49) samples with 100 % specificity, which is good as it is a totally non-invasive method.

Although methylation levels of *DAPK* differed clearly between the Chinese and the Moroccan/Italian samples, we did not observe any additional obvious differences in the methylation patterns between samples from different geographical locations with the current MMSP panel. The Italian and the Chinese samples showed a similar level of MMSP positivity for cellular markers as the Moroccan ones. Thus, an MMSP panel can be applied to samples from different geographical locations with different NPC subtype distribution.

As pattern and timing of methylation status in specific genes are associated with defined biological behaviors [72], we believe that the MMSP method will not only provide diagnostic information but has the potential of predicting behavior of individual tumors, as part of future personalized medicine. Monitoring EBV load and methylation levels of specific genes have been shown to be useful for monitoring disease relapse after treatment, suggesting that MMSP may serve as a way of outcome follow-up after NPC treatment.

It will be meaningful to extend the application of the MMSP assay for the diagnosis of other types of cancer by establishing MMSP patterns with a different panel of marker genes on (tumor) DNA from body fluids, such as detection of prostate cancer or bladder cancer with DNA from urine, cervical cancer by cervical swab, or lung cancer by sputum or blood.

## Conclusions

MMSP can be useful for detection of NPC by a single reaction using a small amount of DNA. It could be developed into a robust, specific, sensitive, and cost-effective screening technique. If applied to nasopharyngeal swabs, or possibly blood/serum, it can be used for the early detection of NPC in high-risk populations. The method is easy to manage in a clinical setting and requires only routine small equipment. Further studies are required to validate the feasibility of the MMSP assay as a population-based screening tool in NPC high-risk populations as well as a way of monitoring tumor recurrence. Detection of the methylation of specific genes has the potential not only to provide diagnostic information but also to provide information about the specific behavior of individual tumors, which could direct diagnostic, preventive, and even therapeutic strategies. Unfortunately, we were unable to demonstrate any feasibility to use serum for the detection of NPC-specific DNA, although the pilot cohort was very small. We showed earlier that nasopharyngeal swabs could be used for this purpose with both better performances. Thus, our modified MMSP assay should also be evaluated on DNA from nasopharyngeal swabs for NPC detection.

## Methods

### Cell lines and clinical samples

Human cell line DNA of CNE1 (EBV negative, NPC [73]) and Namalwa (EBV positive, latency III, Burkitt lymphoma [74, 75]) were cultured in RPMI 1640 medium (Gibco® by life technologies) containing 10 % fetal calf serum (FCS) at 37 °C with 5 % CO_2_. Namalwa has two copies of EBV genome/cell [76]. These two cell lines were used as a positive control for this semi-quantitative methylation PCR. DNA from 44 biopsies from pathology-verified NPC patients and 18 non-cancerous volunteers were obtained from Institut Pasteur du Maroc, Casablanca, Morocco, in the year 2011 (ethical approval: No. 00-302, Stockholm, Sweden, and 2010-02-15, Casablanca, Morocco). Out of 44 NPC samples, 36 samples were positive for *EBNA1* whereas 8 samples were *EBNA1* negative. In 36 *EBNA1* positive samples, 27 were male while 9 were females. The average and median age of the *EBNA1* positive patients was 45 and 50 years, respectively, ranging between 12 and 78 years. The average age of control donors was 26 years. Out of 36 *EBNA1* positive samples, 34 were of NPC type III. NPC DNA samples from 13 matched biopsies and serum samples from pathology-verified NPC patients and additional serum samples from 20 non-cancerous volunteers were obtained from Italy in the year 2012 (ethical approval: No. 00-302, Stockholm, Sweden, and 2010-02-15, Casablanca, Morocco). The samples were stored at −80 °C until further use.

### DNA extraction and conversion by bisulfite modification

DNA was extracted from cell lines, biopsies, and serum and purified by conventional phenol/chloroform and ethanol extraction method. Bisulfite conversion of the DNA was performed by using EZ DNA methylation Kit from Zymo Research (Cat#: D5002) following the protocol. CNE1 and Namalwa DNA were mixed in a 1:2 ratio, respectively, to be used as a positive control.

### Methylation-specific PCR (MSP)

For each PCR reaction, 4 microliters (μl; 40 nanogram (ng)) of bisulfite-modified DNA was added in a final volume of 25 μl of PCR mixture containing 1.8× PCR buffer, 5 millimolar (mM) magnesium chloride (MgCl_2_), 100 picomole (pmol) deoxynucleotide triphosphates, primers (0.1 micromolar (μM) each per reaction), and 2.5 unit of Taq Platinum (Invitrogen). Water was used as the negative control. The MSP primers used were taken from the published data or were designed using the MethPrimer software (Table 6) [77]. The primers for *EBNA1* localized inside the C-terminal coding region [78] and were designed to amplify bisulfite-converted EBV genome without distinguishing between methylated and unmethylated CpGs. Primers for housekeeping gene *GAPDH* served as a quality control for input DNA. This marker could provide information if the bisulfite treatment is complete and if the template is of good quality. Primers for all other markers were inside the CpG-rich promoter region. Primers for *LMP1* were specific to amplify unmethylated bisulfite-converted sequence. Primers for all potential TSGs, i.e., *ITGA9*, *RASSF1A*, *P16*, *DAPK*, *WNT7A*, *CHFR*, *CYB5R2*, *WIF1*, *RIZ1*, and *FSTL1*, were designed to specifically amplify methylated bisulfite-converted sequence. PCR amplifications were performed at 95 °C for 3 min, followed by 4 cycles at 94 °C for 1 min, 60 °C for 30 s, and 65 °C for 45 s, which was then followed by 36 amplification cycles at 94 °C for 1 min, 56 °C for 1 min, and 65 °C for 45 s. It was followed by a final elongation step at 65 °C for 4 min. MSP products were analyzed by 2.5 % agarose gel electrophoresis stained with ethidium bromide.

**Table 6:** Sequence of MSP primers used in the study

Marker		Primer sequence (5′–3′)	Size (bp)	Refs
*EBNA1*	F	AAGAGGTTTAGGAGTTTTAGTAGTTAGTTAT	130	^a^
	R	CACCTTCTTAATAATATTCAAAATAATC	130	
U-*LMP1*	F	GGGGGGATTTGTTTTTTTAATATAAATATAT	145	^a^
	R	TAAAATATAAACCCAAAAAAATTTACA	145	
M-*ITGA9*	F	GTTGTTGGTTCGGAGATTATATTTC	230	^a^
	R	AAAACAACCCGAATAAAAAACG	230	
M-*RASSF1A*	F	GGGTTTTGCGAGAGCGCG	169	[79]
	R	GCTAACAAACGCGAACCG	169	
M-*P16*	F	CGAGTATTCGTTTACGGC	106	^a^
	R	CTTCCTCCGATACTAACG	106	
M-*DAPK*	F	GGATAGTCGGATCGAGTTAACGTC	98	[79]
	R	CCCTCCCAAACGCCGA	98	
M-*CHFR*	F	*GTTTTA*ATATAATATGGCGTCGATC	213	^a^
	R	*C*TCAACTAATCCGCGAAACG	213	
M-*GAPDH*	F	TTAGGTGGTTTTTTTTGATTTTAAT	192	^a^
	R	AAATTATCAAAACCCTTTTTCTAAACCAA	192	
M-*WNT7A*	F	GTAGTTCGGCGTCGTTTTAC	123	[80]
	R	CGAAACCGTCTATCGATACG	123	
M-*CYB5R2*	F	GGGGAGCGGGTTAGTCGTC	140	[81]
	R	GAACCCGCAAACTCGTAACGTC	140	
M-*WIF1*	F	GGGCGTTTTATTGGGCGTATC	198	^a^
	R	*TAACG*AAACCAACAATCAACG	198	
M-*RIZ1*	F	ATTTTAGTTTTAGGGTGCGGTC	233	^a^
	R	AACTCCAATCGAAAATAACGTC	233	
M-*FSTL1*	F	TCGAGGTTGGCGATCGCG	171	[82]
	R	CGCAAACTCGCTCCGACCG	171	

^a^Primers designed in our lab using MethPrimer

### Multiplex methylation-specific PCR

Based on our earlier data on the sensitivity and specificity of several markers, we re-used *EBNA1*, *LMP1*, and *RASSF1A* from the previous MMSP [48] but replaced *DAPK* and *CHFR* with *ITGA9* and *P16*. We also replaced *β-ACTIN* with *GAPDH* to make the newer MMSP assay suitable for both biopsy and serum samples. The *EBNA1* is not regulated by promoter methylation and distinguishes between EBV-positive and EBV-negative samples. EBV-encoded oncogenic *LMP1*—the main EBV transforming protein in NPC—is expressed in 65 % of NPC patients, and this is associated with its promoter methylation status [49]. The presence of *LMP1* in the MMSP panel of genes would provide information about its expression status. The MMSP assay also includes the housekeeping gene *GAPDH* that serves as a quality control for input DNA.

A 1:2 mixture of DNA from CNE1 (EBV negative, NPC) and Namalwa (EBV positive, latency III, Burkitt lymphoma) was used as control, in order to get a positive signal for all our marker genes. The MMSP assay also included the housekeeping gene *GAPDH* as quality control of input DNAs.

For each MMSP PCR reaction, 4 μl (40 ng) of bisulfite-modified DNA was added in a final volume of 25 μg of PCR mixture containing 1.8× PCR buffer, 5 mM MgCl_2_, 0.3 nM deoxynucleotide triphosphates, primers (*ITGA9*: 40 nM, *GAPDH*: 25 nM, *RASSF1A*: 40 nM, *LMP1*: 60 nM, *EBNA1*: 30 nM, and *P16*: 100 nM per reaction), and 2.5 unit of Taq Platinum (Invitrogen). Water was used as the negative control.

The primers and PCR conditions for MMSP amplification and gel electrophoresis were the same as stated for MSP.

## Abbreviations

CHFR: checkpoint with forkhead and ring finger domains
CYB5R2: cytochrome b5 reductase 2
DAPK: death-associated protein kinase
DNA: deoxyribonucleic acid
EBNA1: EBV nuclear antigen 1
EBV: Epstein-Barr virus
FCS: fetal calf serum
FSTL1: follistatin-like 1
GAPDH: glyceraldehyde-3-phosphate dehydrogenase
ITGA9: integrin, alpha 9
LMP1: latent membrane protein-1
MgCl_2_: magnesium chloride
mM: millimolar
MMSP: multiplex methylation-specific PCR
MSP: methylation-specific PCR
ng: nanogram
NPC: nasopharyngeal carcinoma
NPV: negative predictive value
P16: cyclin-dependent kinase inhibitor 2A
pmol: picomole
PPV: positive predictive value
RASSF1A: Ras association (RalGDS/AF-6) domain family member 1
RIZ1: PR domain containing 2, with ZNF domain
RPMI: Roswell Park Memorial Institute medium
TSG: tumor suppressor gene
WHO: World Health Organization
WIF1: WNT inhibitory factor 1
WNT7A: wingless-type MMTV integration site family, member 7A
μl: microliter
μM: micromolar
